# Validation of a Spanish Version of the Physical Appearance Comparison Scales

**DOI:** 10.3390/ijerph17207399

**Published:** 2020-10-11

**Authors:** Cristina Senín-Calderón, José Santos-Morocho, Juan F. Rodríguez-Testal

**Affiliations:** 1 Department of Psychology, University of Cadiz, Puerto Real, 11519 Cadiz, Spain; cristina.senin@uca.es; 2 Department of Psychology, University of Cuenca, 010107 Cuenca, Ecuador; santosjosek1@gmail.com; 3 Personality, Evaluation and Psychological Treatment Department, University of Seville, 41018 Seville, Spain

**Keywords:** appearance comparison, PACS-R, PACS-3, dysmorphic concern, disordered eating

## Abstract

Physical appearance comparison has been widely studied because of its strong relationship with body dissatisfaction and disordered eating. The main objective of this study was to validate the physical appearance comparison scales (PACS-Revised and PACS-3) in a sample of Spanish men and women and examine their psychometric properties. The sample consisted of 1151 participants (age *M* = 22.31, *SD* = 3.40). A unidimensional structure was corroborated in the PACS-R, and three factors in the PACS-3 (proximal, distal, and muscularity comparisons). The PACS-R and PACS-3 showed full scalar invariance across sex. The internal consistency for the PACS-R and subscales of PACS-3 were satisfactory. Positive statistically significant relationships were found with measures of disordered eating (EAT-26) and dysmorphic concern (DCQ). Hierarchical multiple regression analyses demonstrated that the PACS-3 discretely improved the prediction of disordered eating over PACS-R, but did not show improvement in the prediction of dysmorphic concern beyond the PACS-R. These findings suggest that the PACS-R and PACS-3 may be useful tools for evaluating the tendency of men and women to compare their physical appearance.

## 1. Introduction

Body image is a multidimensional construct related to identity which refers to feelings, perceptions, beliefs and behaviors associated with one’s own body [1]. 

The scientific literature on body image has increased noticeably in recent years. In addition to the effects of cultural emphasis on the body and the canons of beauty, part of this increase is related to the impacts that appearance-focused activities on social media such as Facebook or Instagram, have on body image [2]. The use of these social networks, where photographs of idealized bodies abound, has been associated with greater body dissatisfaction [3,4]. This occurs when there is a discrepancy between the ideal and real body assessment (one’s own or social) being compared to [5]. Although body dissatisfaction is not in itself considered pathological [6], it has been associated with depressive symptoms [7], low self-esteem [8], and anxiety [9], and is a risk factor for eating disorders [10]. 

Objectification theory [11], sociocultural theories (tripartite influence model) [12], and others have attempted to explain the development of body dissatisfaction in relation to communication media.

Objectification theory [11] argues that the cultural context marked by gender-role socialization sexualizes the female body, treating it as an object to be observed, admired, and evaluated based on its appearance [11]. Interiorization of this perspective of the observer of one’s own body (self-objectivation), is manifested in greater body vigilance and control, which can lead to body dissatisfaction when it is perceived as not meeting the desired body ideal [13,14]. 

The tripartite influence model [12] is one of the most widely recognized theories attempting to explain body dissatisfaction and disordered eating. This model emphasizes the role of communications media, peers, and equals in transmitting and reinforcing an ideal of beauty in body image that is difficult to achieve. The Western ideal of beauty for men is based on an athletic, slightly muscled body [15], while for women, it has long been based on extreme thinness [12]. However, in recent years the “fitspiration” movement, which promotes not only a thin ideal, but an athletic and toned body as an indicator of health, has been predominating in the social networks [16] and changing the ideal of feminine beauty [17]. According to the tripartite model, body dissatisfaction and disordered eating develop when, due to persistent sociocultural influences, the body image is internalized and the person makes frequent comparisons of their own physical appearance with that of others. Myers et al. [18] found that women who internalized the thin ideal more experienced stronger body image disturbance when they became involved in upward appearance comparisons—that is, with persons considered to be more attractive. Although the tripartite influence model has been studied more widely in women [19], it has also been shown to be applicable to men for explaining body dissatisfaction [20,21]. 

The conceptual development of the comparison based on physical appearance started out from the social comparison theory by Festinger [22]. This theory argues that people often evaluate themselves and tend to compare themselves with others when they do not have objective standards to compare with. People innately compare themselves with others in characteristics which are considered to be relevant [22]. 

Comparisons of physical appearance may have different targets. They may be proximal (i.e., comparison with peers or equals in the social setting in which the person moves) or distal (mainly celebrities) [23]. Appearance comparison can be upward or downward—downward refers to when targets are considered inferior (e.g., people considered less attractive), and is related to greater body satisfaction and positive self-esteem [24]. The targets of upward comparisons have attributes which are considered to be superior or more attractive. Upward-appearance-focused comparisons have been associated with more negative affect, guilt, body checking [25], and low self-esteem [18], and have been shown to be a significant predictor of body dissatisfaction and disordered eating [12,23,26,27], contributing to maintaining body dysmorphic disorder (BDD) symptoms [28]. 

Most of the studies published on appearance comparison have focused their objectives on the relationship with concern for fat/body shape and disordered eating, but few have approached the relationship to dysmorphic concerns or symptoms characteristic of BDD, even though appearance comparison is a frequent behavior of persons affected by this disorder [29,30]. The scarcity of studies on the subject led us to consider it important to determine to what extent appearance comparison can help to predict dysmorphic concern. 

Appearance comparison has mainly been evaluated using self-reports. The most widely used is the Physical Appearance Comparison Scale (PACS) [31], which evaluates its frequency in five items. Schaefer and Thompson [32] published a revision of the scale (PACS-R), because they found some psychometric problems and some items which were more oriented toward female concerns. The PACS-R has been shown to have excellent internal consistency, and its items are written in a neutral manner, with widened evaluation contexts, although the new evaluation contexts only include proximal comparisons. Recently, Schaefer and Thompson [23] broadened the evaluation targets of the scale (PACS-3) to include distal contexts (based on the proximal ones), where comparison of shape/weight, general appearance, and musculature are evaluated. It also evaluates the direction of the comparison (upward or downward) and the emotional impact caused. The latter makes the PACS-3 of greater interest than the Upward and Downward Physical Appearance Comparisons UPACS or DACS scales [33], which along with the PACS are possibly the most widely used to evaluate appearance comparison. The UPACS and DACS scales were the first scales created specifically for evaluating upward and downward physical appearance comparisons, respectively. The UPACS and DACS have only been validated in the Chinese cultural context, where their psychometric properties were demonstrated to be adequate [34]. However, Schaefer and Thompson [32] criticized some of the items in the UPACS and DACS because they evaluate the comparison of appearance based on stereotypes of attractiveness—for example, assuming that an overweight body is not attractive.

In view of the importance of evaluating appearance comparison, there is a pressing need for validated measures in languages other than English by independent research groups in different cultural contexts, in order to be able to continue progressing in the study of body image disturbance. As far as we know, there is no validated measure in Spanish which evaluates the comparison of appearance, and can therefore test a model as widely studied as the tripartite influence model, where physical appearance comparisons act as the mediating variable between sociocultural influences and body dissatisfaction and body image disturbance [35]. The validation of these scales could extend their use to other Spanish-speaking countries with their corresponding cultural adaptations, enabling wider knowledge of body dissatisfaction in other populations, since the study of body image has been based mainly on US, British, and Australian samples [36].

Thus, this study proposes the validation of the Spanish PACS-R and PACS-3 scales with the following specific objectives: (1) Validate the factor structure of the PACS-R and PACS-3 in a sample of young Spaniards; (2) Examine their invariance of measurement across sex; (3) Find their internal consistency and evidence of concurrent validity; and (4) Analyze the incremental validity of the PACS-3 over the PACS-R for predicting disordered eating and dysmorphic concern. 

## 2. Materials and Methods 

### 2.1. Participants

The sample was comprised of 1151 subjects from the South of Spain (11.12% Psychology students), of whom 55.4% were women. The mean age was 22.31 years (*SD* = 3.40, range 18 to 35) and BMI from 15.43 to 48.44 kg/m^2^ (*M* = 23.32, *SD* = 4.33). The inclusion criteria were age 18 to 35 and user of the Instagram social network. The latter was considered because validation of the PACS-R and PACS-3 is part of a wider project evaluating the use of this social network and body image disturbance. 

### 2.2. Measures

Before the evaluation tests were administered, the participants completed a sociodemographic questionnaire (sex, age, weight, and height).

Physical Appearance Comparison Scale-Revised (PACS-R) [32]. Eleven items evaluate the frequency, from 0 “Never” to 4 “Always”, with which participants compare their physical appearance with other persons in different social situations (e.g. “When I’m with a group of friends, I compare my body size to the body size of others”). Comparison targets are proximal. The authors of the scale found excellent internal consistency (Cronbach’s α = 0.97) and convergent validity with measures of body satisfaction, eating pathology, and sociocultural influence in appearance and self-esteem. For the Spanish translation of the PACS-R, see Table A1.

Physical Appearance Comparison Scale–3 (PACS-3) [23]. This scale has 27 items which evaluate the comparison of general physical appearance, weight/shape, and musculature (e.g., “When I watch a movie, I compare my overall appearance to the appearance of the actors/actresses”; “When I see a model in a magazine, I compare my weight/shape to his/her weight/shape”; “When I’m out in public, I compare my muscularity to the muscularity of others). The scale consists of three factors: proximal, distal, and muscular comparison. Nine items evaluate the frequency of comparison, nine items evaluate the direction of the comparison (upward/downward), and nine items the emotional impact (effect) caused by the comparison. The total scale comprises nine subscales: Proximal (frequency, direction, and effect), Distal (frequency, direction, and effect), and Muscularity (frequency, direction, and effect). The answer format is a Likert-type scale in a range of 1 “Never” to 5 “Almost always”. The direction and emotional impact of the comparison are only evaluated if the subject scores 2 to 5 on the items which evaluate comparison frequency. The authors found an internal consistency of Cronbach’s alpha α = 0.76 to 0.91 for the three factors, and from 0.91 to 0.94 for the total scores on frequency, direction, and effect. See Table A2 for the Spanish translation of the PACS-3.

Dysmorphic Concern Questionnaire (DCQ) [37,38]. The DCQ is made up of seven items which evaluate concern for some defect in physical appearance, representing a screening measure for body dysmorphic disorder (e.g., “Spend a lot of time covering up defects in your appearance/bodily functioning”). Items are rated on a four-point Likert-type scale from 0 (“Not at all”) to 3 (“Much more than most people”). Its internal consistency is adequate (Cronbach’s α = 0.88). In a general Spanish population, it had an α = 0.85, test–retest reliability of *e* = 0.87 (average one-month interval), and convergent validity with other measures for detecting dysmorphic concerns [38]. In this study sample, the α was 0.86.

Eating Attitudes Test (EAT-26) [39,40]. This is a screening measure which evaluates symptoms and characteristics of disordered eating (e.g., “Avoid eating when I am hungry”). It is made up of 26 items and three factors: dieting, bulimia and food preoccupation, and oral control. The answer format is a six-point Likert-type scale from 1 “Never” to 6 “Always”. A total score may be found by recoding the answers: from 1 to 3 are recoded as 0, 4 is recoded as 1, 5 as 2, and 6 is recoded as 3. The authors found a Cronbach’s α of 0.83 to 0.90. A unidimensional structure was found for the Spanish validation and internal consistency was 0.90. The scale has demonstrated good specificity and moderate sensitivity in detecting eating disorder [40]. In this sample, the α was 0.88 for the total scale.

### 2.3. Procedure

Permission for a Spanish translation and validation was requested from the authors of the PACS-R and PACS-3 scales. After receiving permission from the authors, the PACS-R and PACS-3 scales were back-translated into Spanish by two bilingual Spanish-English speakers, familiar with the US and Spanish cultures. First, a bilingual Spanish-English speaker translated the scale into Spanish, and then the translated version was translated into English by the other bilingual translator. Any discrepancies between the original scales and the back-translated versions were discussed by the translators with the corresponding author of this manuscript. The final versions of the back-translated scales were administered to 11 students in their last year of study for a degree in Psychology (n = 7 women, 4 men) to verify the understandability of the items and scale instructions, as well as familiarity with the contexts and situations in the items on the scales. The students stated that both scale instructions and items were understandable and referred to everyday situations. 

The scales were filled out in an anonymous online form with an alphanumerical participant identification code. Before filling out the questionnaires, information about the study and an informed consent form were provided. The subjects had to click on consent acceptance to be able to take the tests. First, the tests were taken by university students studying for a degree in Psychology who were rewarded with points in one of their courses. These students, using snowballing techniques, had to administer the tests to five persons who were not Psychology students. The tests were filled out from March 2018 to May 2019. Data from the PACS-3 were collected starting in February 2019 because the scale was not published until the end of 2018. This test was administered to 506 subjects who had previously taken the PACS-R. 

All procedures performed in studies involving human participants were in accordance with the ethical standards of the Andalusian Regional Government Ethics Committee (reference: PACS-2019) and with the 1964 Helsinki declaration and its later amendments or comparable ethical standards.

### 2.4. Data Analysis

Descriptive analyses were performed for the items on the PACS-R and PACS-3 scales. Multivariate normality was checked by the Mardia test. The models found with confirmatory factor analysis (CFA) of the PACS-3 and PACS-R with robust maximum likelihood and the asymptotic covariance matrix were shown to be adequate. The following goodness-of-fit indicators were used: Satorra–Bentler chi square (SBχ^2^); the comparative fit index (CFI); the non-normed fit index (NNFI), which had to be over 0.90 [41]; root mean square error of approximation (RMSEA) and its 90% confidence interval; and the standardized root mean square residual (SRMR), which must be below 0.05 to be considered adequate, and between 0.05 and 0.08 to be considered acceptable [42].

The reliability of the total PACS-R and the PACS-3 subscales was assessed using the Cronbach’s alpha coefficient. Evidence of internal structure validity was estimated using Pearson’s correlation coefficient. The size of the correlation was evaluated by the method described by Cohen [43], in which *r*-values ≥ 0.10, 0.30, and 0.50 are used as benchmarks for small, medium, and large effects, respectively.

Invariance of measurement of the PACS-3 and PACS-R was tested in successive multi-group CFAs to determine if both measures were equivalent across sex. First, a CFA was performed for men and another for women separately. Then, the configural invariance model was tested, in which all parameters were freely estimated across sex. Next, the metric invariance was obtained, where factor loadings were constrained to be equal across sex. Finally, scalar invariance was established, with factor loadings and thresholds constrained to be equal across groups. For comparison of the nested models, the Cheung and Rensvold [44] criterion was used, in which the change in CFI must be below 0.01. 

Finally, a hierarchical multiple regression analysis was performed to determine whether the PACS-3 improved over the predictive qualities in disordered eating as measured by the EAT-26 and dysmorphic concern above and beyond PACS-R, controlling for BMI and sex. In Step 1, BMI and sex were entered as predictor variables. The PACS-R was entered at Step 2. In Steps 3, 4, and 5, the PACS-3 total frequency, direction, and effect were entered, respectively. The changes in *R*^2^ in Steps 3, 4, and 5 were indicators of the incremental validity of the PACS-3. All statistical analyses were performed with Factor 10.4.01, SPSS 24 (IBM Corp., Armonk, NY, USA) and LISREL 8.7 software (Scientific Software International, Lincolnwood, IL, USA).

## 3. Results

### 3.1. Descriptive Analysis of the Items on the PACS-R and PACS-3 Scales

Means, standard deviations, skewness, and kurtosis are presented in Tables S1 and S2 of the Supplementary Material. The skewness and kurtosis of the items were not especially high, although the Mardia test was statistically significant (PACS-R: 182.70, *p* < 0.001; PACS-3: 136.39, *p* < 0.001), showing that the assumption of multivariate normality was not met. 

On the PACS-3 scale, most of the participants answered affirmatively about the frequency with which they normally compared their physical appearance with others, and therefore answered the items related to the direction and emotional impact of the comparison. 

### 3.2. Evidence of PACS-R and PACS-3 Internal Structure Validity 

**Figure 1:**
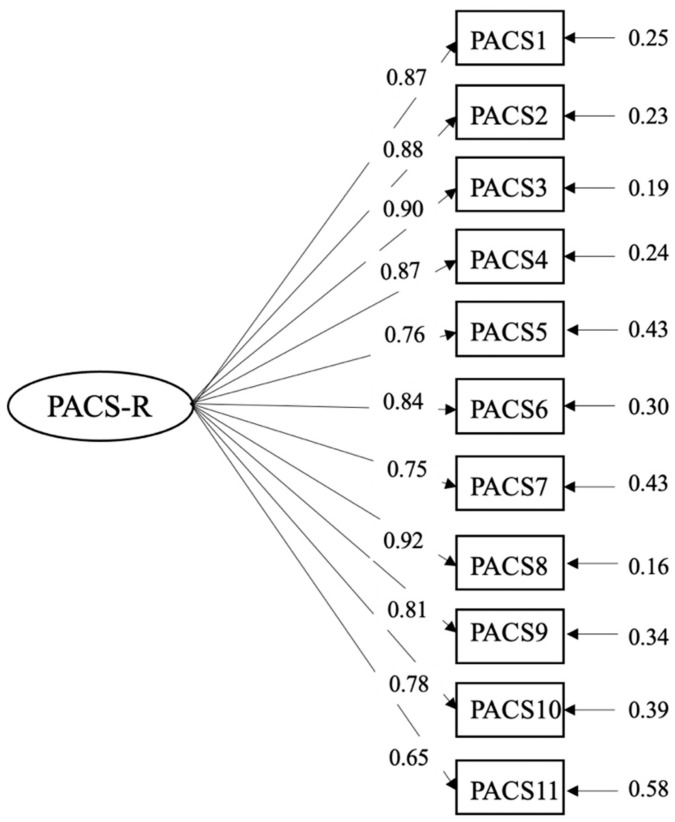
Path diagram and factor loadings of the Physical Appearance Comparison Scale-Revised (PACS-R).

**Figure 2:**
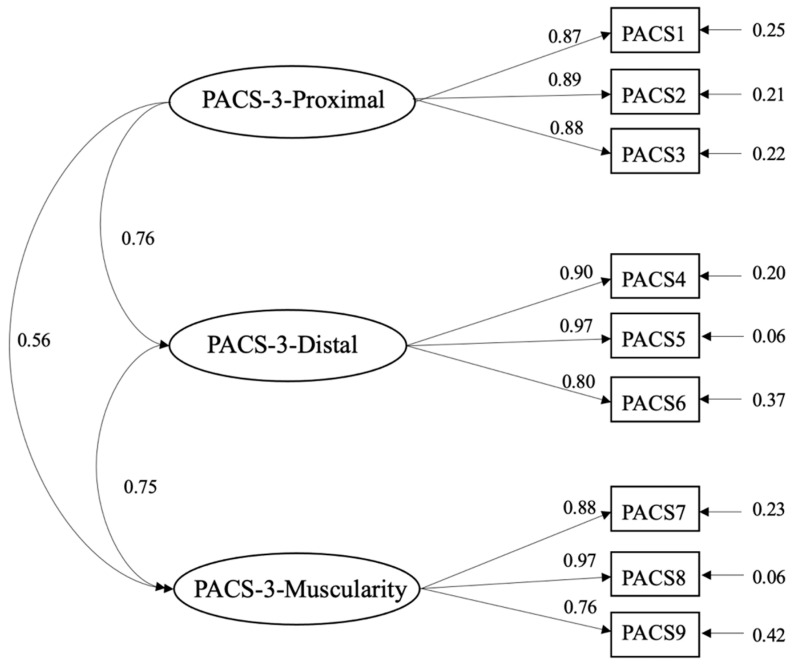
Path diagram and factor loadings of the Physical Appearance Comparison Scale–3 (PACS-3).

A CFA of the PACS-R and PACS-3 was done. The unidimensional structure of the PACS-R was corroborated with adequate goodness-of-fit indicators: SBχ^2^ = 685.29, *df* = 44; NNFI =0.970, CFI = 0.980; SRMR = 0.047, RMSEA = 0.11, 90% CI (0.110, 0.120). The CFA of the PACS-3 confirmed the three-factor structure (proximal, distal, and muscular), finding the following goodness-of-fit indicator values: SBχ^2^ = 147.41, *df* = 24, NNFI = 0.97, CFI = 0.98, SRMR = 0.054, RMSEA= 0.098 (0.083, 0.110). On both scales, the RMSEA was over the recommended values, although this is common when the degrees of freedom are less than 50 [45]. Figure 1 and Figure 2 show the completely standardized factor loadings.

### 3.3. Measurement Invariance of PACS-R and PACS-3

The goodness-of-fit indexes of the models for women and men separately were adequate. The configural invariance in the PACS-R and PACS-3 was proved to have good model fit, showing that the factor structure and loading pattern are equivalent for men and women. The results of the metric invariance showed a ΔCFI < 0.01 and appropriate goodness-of-fit indicators, so both men and women interpreted and answered the items on both scales similarly. Finally, support was found for scalar invariance, with a ΔCFI of < 0.01 in both the PACS-R and PACS-3, showing that the observed scores are related to latent scores (Table 1). 

### 3.4. Reliability and Evidence of Validity Based on Relationships with Measures of Other Variables 

The internal consistency is shown for the PACS-3 subscales and the total PACS-R scale in Table 2. The Cronbach’s α for the subscales varied from 0.82 (muscularity:direction) and 0.90 (distal and proximal:effect). The internal consistency of the PACS-R was α = 0.94. The retest reliability was found for the latter after a time lapse of three weeks *r* = 0.808 (*n* = 339).

Convergent validity was demonstrated by a strong positive correlation between the EAT-26 and PACS-R tests, and between the latter and the DCQ. The correlations between the PACS-3 subscales and the EAT-26 and DCQ were small and moderate. Most of the PACS-3 subscales showed a positive strong or moderate association with the PACS-R. 

The means of the PACS-3 factors and the total PACS-R scale were found for the sexes separately. In general, women had higher averages except in frequency of comparison of musculature. 

### 3.5. Incremental Validity of Disordered Eating and Dysmorphic Concern

Table 3 shows the two hierarchical multiple regression analyses over the disordered eating as measured by the EAT-26 and dysmorphic concern criteria. In Step 1, the predictor variables BMI and sex contributed very little to the explanation of disordered eating *F* (2, 481) = 12.12, *p* < 0.001 and dysmorphic concern *F* (2, 481) = 8.68, *p* < 0.001. By entering PACS-R in Step 2, an additional 26% of the variance in disordered eating *F* (1, 480) = 177.97, *p* < 0.001 and 31% in dysmorphic concern *F* (1, 480) = 230.42, *p* < 0.001 were explained. In Step 3, PACS-3 total frequency predicted the only variance in disordered eating *F* (1, 479) = 6.58, *p* ˂ 0.05 with a contribution of 0.01. However, it did not predict dysmorphic concern *F* (1, 479) = 2.51, *p* > 0.05. In Step 4, the PACS-3 total direction did not explain unique variance in either disordered eating *F* (1, 478) = 0.13, *p* > 0.05 or dysmorphic concern *F* (1, 478) = 2.48, *p* > 0.05. In Step 5, the PACS-3 total effect predicted the only variance in disordered eating with an increase of 0.01 *F* (1, 477) = 9.80, *p* ˂ 0.01 but not dysmorphic concern *F* (1, 477) = 1.89, *p* > 0.05. 

## 4. Discussion

The main objective of this study was to validate Spanish versions of the PACS-R and PACS-3 scales in a sample of young Spaniards. Our objective of examining the validity of the construct of both scales was met by the confirmatory factor analyses with adequate goodness-of-fit indexes corroborating the unidimensional structure of the PACS-R found by the authors [32], and the three-factor structure of the PACS-3 also as found by Schaefer and Thompson [23].

The internal consistency of the total PACS-R was excellent, and the test–retest reliability supported the temporal stability of the scale three weeks after its first application. The scale showed a strong positive relationship with the disordered eating and dysmorphic concern evaluation measures. The PACS-3 subscales had a favorable internal consistency, with alphas over 0.80. Moderate-to-strong relationships were found between the PACS-3 and the PACS-R. The relationships between the PACS-3 subscales with the EAT-26 and DCQ scales were all statistically significant, but moderate in size, and stronger in the proximal comparisons. The relationship between the comparison of musculature, the DCQ, and EAT-26 seems to be less solid. This result may be explained by the characteristics of the participants, and a drive for muscularity or compulsive pursuit of muscularity rather than specific indicators of muscle dysmorphia [46]. In fact, some results have shown that the relationship between eating disorders and these types of behaviors, more related to an interest in muscular training, is moderate [47], similar to the results found here. Finally, it has been suggested that clinically important indicators of muscle dysmorphia would be accompanied by considerable alterations in eating behavior related to bodybuilding [46].

In general, both the PACS-R and the PACS-3 showed evidence of convergent validity. The relationships between appearance comparison and disordered eating are consistent with the findings of Bailey and Ricciardelli [26], Schaefer and Thompson [23,32], and O’Brien et al. [33], while the relationship with dysmorphic concern is backed by studies which have found a relationship between BDD symptoms and appearance comparison [4,28,29,48]. 

As reported by Fardouly, Pinkus, and Vartanian [49] and Fardouly and Vartanian [50], women compared themselves more often with proximal targets than distal, and the same was true of men. However, women made more upward comparisons than men, accompanied by a more negative emotional impact as a result of those comparisons—similar to the findings of Strahan, Wilson, Cressman, and Buote [51]. In line with the contributions of Schaefer and Thompson [23], in general, women participated in more appearance comparisons than men, both with distal and proximal targets. As it is a traditionally more frequent behavior in women, many studies have approached this construct exclusively with women [18,27,32]. However, the current sociocultural trend for an athletic body in women and sociocultural pressures for men to also have an ideal body may lessen these differences, and therefore it was considered relevant to evaluate invariance on both scales. The results showed equivalence between men and women in the construction of the scale and interpretation of the items. 

In agreement with the findings of Bailey and Ricciardelli [26], appearance comparison predicted disordered eating. Multiple regression analyses demonstrated that the PACS-3 improved the prediction of disordered eating by 2%. These results do not coincide with the findings by the authors of the scale [23], who found a much higher predictive power for PACS-3 for disordered eating and body satisfaction than other appearance comparison scales. However, the authors did not use the PACS-R in the prediction, but its predecessor (PACS), which had problems as mentioned in the Introduction. With respect to prediction of dysmorphic concern, the PACS-3 subscales only provided a 1% increase in the variance. The PACS-R had a predictive power for dysmorphic concern of over 30% of the variance. These findings are consistent with the study by Boroughs et al. [48], who found that appearance comparison was the predictor which explained the most single variance in body dysmorphic disorder (BDD) symptomatology in a set of variables related to satisfaction with appearance and body parts, self-esteem, and obligatory exercise. Lambrou et al. [29] found that appearance comparison was the most common behavior of BDD patients. Considering the findings of our study, we cannot conclude that in the sample analyzed the PACS-3 is more advantageous than the PACS-R for predicting disordered eating and dysmorphic concern. 

Based on the results reported, we recommend the use of the PACS-R for evaluating the frequency of appearance comparison in peer contexts. As it is a brief instrument with adequate reliability which has demonstrated strong predictive power for disordered eating and dysmorphic concern, it could be useful in the scope of research and clinical evaluation of this construct. However, the unidimensionality of the scale could be a limitation if it were desired to make a specific evaluation of the construct, and in that case it would be preferable to use the PACS-3. This scale offers a variety of contexts along with the possibility of directly evaluating comparison of muscularity (not only physical appearance, shape, and weight, as in the PACS-R), upward or downward directionality, and the emotional impact caused by the comparison. These characteristics make the PACS-3 a very complete evaluation measure of physical appearance comparison. Furthermore, the inclusion of items that evaluate the comparison of muscularity may be of interest in the evaluation of this comparative tendency in women due to the recent change in the ideal of beauty toward an athletic body. 

For future improvement in appearance comparison scales, we suggest that distal comparison targets closer to young people, such as Instagrammers, YouTubers, bloggers, and influencers, could be included, and in fact anyone they might admire for their attributes and who form part of a digital media consumption, such as social networks or video platforms like YouTube. Media with static content such as magazines will probably disappear from the preferences of young people or at least be used only by a minority, and therefore the items that refer to comparison with models in magazines could lack interest. In support of this argument, Fardouly et al. [49] found that women compared their appearance more on social networks, where they also made more upward comparisons than in the traditional media. Comparison on social networks also generated more body dissatisfaction than comparisons in real social situations. However, these authors also found that women made comparisons in person more often than though other media. This argument supports the usefulness of the PACS-R and PACS-3 scales for evaluating social comparison in real situations since it has several different contexts in which subjects can recognize that they are performing this cognitive process. 

This study has some limitations which should be kept in mind. It is a cross-sectional study of a single measure. Part of the sample did not fill out the PACS-3 because the instrument was published months after data had begun to be collected for the PACS-R, which could not be administered again for retest reliability. The study sample may not be representative of the general population, first because the study participation age was limited (18 to 35), and second because snowball sampling was used with Psychology students as the main sample recruiters, so the evaluation tests were mainly administered to other students in other degree programs. Another limitation is that variables were not included that may have been relevant in the prediction of disordered eating and dysmorphic concern, such as hours of exercise or dieting. Neither were evaluation tests administered that could provide evidence of discriminant validity. However, the study has some strengths. As far as we know, it is the first study which provides a Spanish validation of the appearance comparison construct measures with men and women. Having these evaluation scales is important in the scope of psychological intervention in body image disturbance. Recently, McLean et al. [52] found considerable sustained improvements in body satisfaction, internalization of thinness, and fear of negative evaluation of appearance in teenage girls by applying an intervention program focusing exclusively on appearance comparison. Another relevant contribution of this study is the finding of invariance of measurement across sex of the PACS-R and PACS-3, which was unknown until now. The authors validated the PACS-R only with women, and even though the PACS-3 was filled out by men and women, no invariance analysis was provided in the validation study. Furthermore, novel results are provided on the predictive power of appearance comparison on dysmorphic concern. Perhaps in the future, the relationship between appearance comparison and muscular dysmorphia would also need to be evaluated. 

## 5. Conclusions

Both PACS-R and PACS-3 scales are reliable and valid measures for assessing the physical appearance comparison, both for men and women. We recommend using PACS-R for a screening assessment. For a more complete evaluation, PACS-3 should be used. The PACS-3 discretely improved the prediction of disordered eating over PACS-R, but did not show improvement in the prediction of dysmorphic concern beyond the PACS-R.

## Figures and Tables

**Table 1:** Goodness-of-fit indexes for tested invariance of the PACS-3 and PACS-R across sex.

	**PACS-3**						
	**SBχ^2^**	***df***	**CFI**	**NNFI**	**RMSEA**	**SRMR**	**∆CFI**
Men (*n* = 263)	112.08	24	0.97	0.96	0.11 (0.09, 0.14)	0.08	-
Women (*n* = 244)	61.68	24	0.99	0.98	0.08 (0.05, 0.10)	0.04	-
Configural	172.83	48	0.98	0.97	0.10 (0.08, 0.12)	0.12	-
Metric	218.76	54	0.98	0.97	0.11 (0.09, 0.12)	0.15	0.004
Scalar	252.35	60	0.97	0.97	0.11 (0.09, 0.12)	0.13	0.004
	**PACS-R**						
	**SBχ^2^**	***df***	**CFI**	**NNFI**	**RMSEA**	**SRMR**	**∆CFI**
Men (*n* = 513)	370.062	44	0.970	0.962	0.12 (0.109, 0.132)	0.066	-
Women (*n* = 640)	367.641	44	0.981	0.976	0.10 (0.097, 0.118)	0.042	-
Configural	674.86	90	0.980	0.970	0.11 (0.101, 0.120)	0.070	-
Metric	808.76	98	0.974	0.971	0.11 (0.105, 0.119)	0.081	0.006
Scalar	770.536	110	0.976	0.976	0.10 (0.095, 0.109)	0.108	−0.002

Note: PACS-3: Physical Appearance Comparison Scale; SBχ^2^: Satorra–Bentler chi square; df: degrees of freedom; CFI: Comparative Fit Index; NNFI: Non-Normed Fit Index; RMSEA: Root Mean Square Error of Approximation; SRMR: Standardized Root Mean Square Residual; PACS-R: Physical Appearance Comparison Scale Revised (PACS-R).

**Table 2:** Pearson correlation coefficient, reliability, and descriptive statistics for PACS-3 and PACS-R.

	PACS-3 Proximal			PACS-3 Distal			PACS-3 Muscularity			PACS-3 Total			PACS-R
**Pearson Correlation**													
	**F ^a^**	**D ^b^**	**E ^c^**	**F**	**D**	**E**	**F**	**D**	**E**	**F**	**D**	**E**	
**PACS-R**	0.57 **	0.41 **	0.43 **	0.45 **	0.39 **	0.40 **	0.31 **	0.27 **	0.30 **	0.52 **	0.46 **	0.47 **	-
**EAT-26**	0.38 **	0.31 **	0.34 **	0.34 **	0.32 **	0.34 **	0.21 **	0.18 **	0.24 **	0.36 **	0.32 **	0.36 **	0.58 **
**DCQ**	0.41 **	0.35 **	0.36 **	0.32 **	0.24 **	0.26 **	0.25 **	0.19 **	0.23 **	0.39 **	0.34 **	0.36 **	0.60 **
**BMI**	0.07	0.15 **	0.15 **	0.01	0.06	0.08	−0.03	−0.060	−0.02	0.021	0.06	0.07	0.13
**Reliability**													
**α**	0.88	0.87	0.90	0.89	0.88	0.90	0.88	0.82	0.84	0.91	0.91	0.93	0.94
**Mean (*SD*)**													
***M (SD)* women**	3.07 (1.00)	3.30 (0.84)	3.25 (0.89)	2.66 (1.12)	3.47 (0.98)	3.22 (0.97)	2.12 (1.03)	3.08 (1.05)	2.91 (1.03)	2.62 (0.91)	2.91 (1.05)	2.77 (1.04)	1.75 (0.96)
***M (SD)* men**	2.52 (0.98)	2.84 (0.98)	2.75 (0.98)	2.11 (0.89)	2.94 (1.05)	2.65 (0.96)	2.31 (0.99)	2.84 (1.13)	2.63 (1.05)	2.32 (0.82)	2.60 (1.04)	2.43 (1.05)	1.21 (0.84)

Note: PACS-3: Physical Appearance Comparison Scale 3; PACS-R: Physical Appearance Comparison Scale-Revised; EAT-26: Eating Attitudes Test; DCQ: Dysmorphic Concern Questionnaire; BMI: Body Mass Index; α: Cronbach´s α; ^a^ F: frequency, ^b^ D: direction, ^c^ E: emotional effect. ** *p* < 0.01.

**Table 3:** Summary hierarchical regression analysis predicting disordered eating and dysmorphic concern.

	Disordered Eating			Dysmorphic Concern		
Predictors	*R* ^2^	∆*R*^2^	*β*	*R* ^2^	*∆R* ^2^	*β*
Step 1	0.04	0.05 **		0.03	0.03 **	
BMI			0.19 **			0.06
Sex			0.13 **			0.18 **
Step 2	0.30	0.26 **		0.34	0.31 **	
BMI			0.09 *			−0.05
Sex			−0.04			−0.01
**PACS-R**			00.54 **			0.60 **
Step 3	0.31	0.01 *		0.35	0.01	
BMI			0.10 *			−0.04
Sex			−0.04			−0.01
PACS-R			0.48 **			0.56 **
**PACS-3 Frequency**			0.11 *			0.07
Step 4	0.31	0.00		0.35	0.01	
BMI			0.09 *			−0.05
Sex			−0.04			−0.01
PACS-R			0.48 **			0.56 **
PACS-3 Frequency			0.10			−0.00
**PACS-3 Direction**			0.02			0.09
Step 5	0.32	0.01 **		0.35	0.01	
BMI			0.08*			−0.05
Sex			−0.05			−0.02
PACS-R			0.48 **			0.55 **
PACS-3 Frequency			0.06			−0.02
PACS-3 Direction			−0.33 *			−0.06
**PACS-3 Effect**			0.42 **			0.18

Note: BMI: Body Mass Index, *β*: standardized beta weight. * *p* <0.05. ** *p* < 0.01.

## Supplementary Materials

The following are available online at https://www.mdpi.com/1660-4601/17/20/7399/s1, Table S1: Descriptive statistics of items on the PACS-R (*n* = 1151), Table S2: Descriptive statistics of items on the PACS-3 (*n* = 506).

## Appendix A

**Table A1:** Spanish translation of the Physical Appearance Comparison Scale-Revised (PACS-R).

Ítems	Respuesta				
1. Cuando estoy en público, comparo mi apariencia física con la de los demás	0	1	2	3	4
2. Cuando conozco a una persona nueva (de mi mismo sexo), comparo el tamaño de mi cuerpo con el suyo	0	1	2	3	4
3. Cuando estoy en el trabajo o en el instituto o universidad comparo la forma de mi cuerpo con la de los demás	0	1	2	3	4
4. Cuando estoy en público, comparo mi grasa corporal con la de los demás	0	1	2	3	4
5. Cuando estoy comprando ropa, comparo mi peso con el de los demás	0	1	2	3	4
6. Cuando estoy en una fiesta, comparo la forma de mi cuerpo con la de los demás	0	1	2	3	4
7. Cuando estoy con mi grupo de amigos, comparo mi peso con el de los demás	0	1	2	3	4
8. Cuando estoy en público, comparo el tamaño de mi cuerpo con el de los demás	0	1	2	3	4
9. Cuando estoy con mi grupo de amigos, comparo mi tamaño corporal con el de los demás	0	1	2	3	4
10. Cuando estoy comiendo en un restaurante o lugar público, comparo mi grasa corporal con la de los demás	0	1	2	3	4
11. Cuando estoy en el gimnasio, comparo mi apariencia física con la de los demás	0	1	2	3	4

La gente a veces compara su apariencia física con la de los demás. Puede tratarse de una comparación de su peso, tamaño, forma, grasa corporal o apariencia general. Piense en cómo se compara normalmente con otros. Por favor, use la siguiente escala para calificar la frecuencia con la que realiza este tipo de comparaciones. 0 = Nunca; 1 = Raramente; 2 = Algunas veces; 3 = A menudo 4 = Siempre.

**Table A2:** Spanish translation of the Physical Appearance Comparison Scale–3 (PACS-3).

Ítems	Respuesta				
	1	2	3	4	5
1) Cuando estoy en una fiesta o reunión social, comparo mi apariencia general con la de los demás.	Nunca	Rara vez	A veces	A menudo	Casi siempre
1b) Cuando hago estas comparaciones, generalmente creo que me veo ___________ que la persona con la que me estoy comparando.	Mucho mejor	Mejor	Igual	Peor	Mucho peor
1c) Cuando haces estas comparaciones, ¿cómo te hace sentir normalmente?	Muy positivo	Positivo	Neutro	Negativo	Muy negativo
2) Cuando estoy en público, comparo mi peso/forma con el de los/as demás.	Nunca	Rara vez	A veces	A menudo	Casi siempre
2b) Cuando hago estas comparaciones, generalmente creo que me veo ___________ que la persona con la que me estoy comparando.	Mucho mejor	Mejor	Igual	Peor	Mucho peor
2c) Cuando haces estas comparaciones, ¿cómo te hace sentir normalmente?	Muy positivo	Positivo	Neutro	Negativo	Muy negativo
3) Cuando me encuentro con una persona nueva (del mismo sexo), comparo mi peso/forma con su peso/forma.	Nunca	Rara vez	A veces	A menudo	Casi siempre
3b) Cuando hago estas comparaciones, generalmente creo que me veo ___________ que la persona con la que me estoy comparando.	Mucho mejor	Mejor	Igual	Peor	Mucho peor
3c) Cuando haces estas comparaciones, ¿cómo te hace sentir normalmente?	Muy positivo	Positivo	Neutro	Negativo	Muy negativo
4) Cuando veo una película, comparo mi apariencia general con la de los actores/actrices.	Nunca	Rara vez	A veces	A menudo	Casi siempre
4b) Cuando hago estas comparaciones, generalmente creo que me veo ___________ que la persona con la que me estoy comparando.	Mucho mejor	Mejor	Igual	Peor	Mucho peor
4c) Cuando haces estas comparaciones, ¿cómo te hace sentir normalmente?	Muy positivo	Positivo	Neutro	Negativo	Muy negativo
5) Cuando veo la televisión, comparo mi peso/forma con el de los actores/actrices.	Nunca	Rara vez	A veces	A menudo	Casi siempre
5b) Cuando hago estas comparaciones, generalmente creo que me veo ___________ que la persona con la que me estoy comparando.	Mucho mejor	Mejor	Igual	Peor	Mucho peor
5c) Cuando haces estas comparaciones, ¿cómo te hace sentir normalmente?	Muy positivo	Positivo	Neutro	Negativo	Muy negativo
6) Cuando veo un/a modelo en una revista, comparo mi peso/forma con su peso/forma.	Nunca	Rara vez	A veces	A menudo	Casi siempre
6b) Cuando hago estas comparaciones, generalmente creo que me veo ___________ que la persona con la que me estoy comparando.	Mucho mejor	Mejor	Igual	Peor	Mucho peor
6c) Cuando haces estas comparaciones, ¿cómo te hace sentir normalmente?	Muy positivo	Positivo	Neutro	Negativo	Muy negativo
7) Cuando veo un/a modelo en una revista, comparo mi musculatura con la suya.	Nunca	Rara vez	A veces	A menudo	Casi siempre
7b) Cuando hago estas comparaciones, generalmente creo que me veo ___________ que la persona con la que me estoy comparando.	Mucho mejor	Mejor	Igual	Peor	Mucho peor
7c) Cuando haces estas comparaciones, ¿cómo te hace sentir normalmente?	Muy positivo	Positivo	Neutro	Negativo	Muy negativo
8) Cuando veo una película, comparo mi musculatura con la de los actores/actrices.	Nunca	Rara vez	A veces	A menudo	Casi siempre
8b) Cuando hago estas comparaciones, generalmente creo que me veo ___________ que la persona con la que me estoy comparando.	Mucho mejor	Mejor	Igual	Peor	Mucho peor
8c) Cuando haces estas comparaciones, ¿cómo te hace sentir normalmente?	Muy positivo	Positivo	Neutro	Negativo	Muy negativo
9) Cuando estoy en público, comparo mi musculatura con la de los demás.	Nunca	Rara vez	A veces	A menudo	Casi siempre
9b) Cuando hago estas comparaciones, generalmente creo que me veo ___________ que la persona con la que me estoy comparando.	Mucho mejor	Mejor	Igual	Peor	Mucho peor
9c) Cuando haces estas comparaciones, ¿cómo te hace sentir normalmente?	Muy positivo	Positivo	Neutro	Negativo	Muy negativo

La gente a veces compara su apariencia física con la de los demás. Puede tratarse de una comparación de su peso o forma, musculatura o de su apariencia general. A continuación, encontrará una lista de diferentes contextos en los que las personas pueden realizar este tipo de comparaciones de su apariencia física. Para cada tipo de comparación, por favor haga lo siguiente: Paso 1: Primero indique con qué frecuencia realiza este tipo de comparaciones (utilizando la escala proporcionada, de “nunca” a “casi siempre”). Paso 2: Si nunca hace el tipo de comparación en particular (es decir, califica el ítem como “Nunca”), entonces vaya directamente al siguiente conjunto de ítems. Sin embargo, si califica un ítem como “Rara vez”, “A veces”, “A menudo” o “Casi siempre”, califique también cómo se sintió en relación con el objetivo de la comparación (de “mucho mejor” a “mucho peor”), y cómo le hizo sentir esa comparación ( de “muy positivo” a “muy negativo”).

## References

[1] Cash T.F., Pruzinsky T. (1990). Body Images: Development, Deviance and Change.

[2] Saiphoo A.N., Vahedi Z. (2019). A meta-analytic review of the relationship between social media use and body image disturbance. Comput. Hum. Behav..

[3] Holland G., Tiggemann M. (2016). A systematic review of the impact of the use of social networking sites on body image and disordered eating outcomes. Body Image.

[4] Senín-Calderón C., Perona-Garcelán S., Rodríguez-Testal J.F. (2020). The dark side of Instagram: Predictor model of dysmorphic concerns. Int. J. Clin. Health Psychol..

[5] Grogan S. (2008). Body Image: Understanding Body Dissatisfaction in Men, Women, and Children.

[6] Cash T.F., Cash T.F., Pruzinsky T. (2002). A negative body image: Evaluating epidemiological evidence. Body Image: A Handbook of Theory, Research, and Clinical Practice.

[7] Senín-Calderón C., Rodríguez-Testal J.F., Perona-Garcelán S., Perpiñá C. (2017). Body image and adolescence: A behavioral impairment model. Psychiatry Res..

[8] Wichstrøm L., von Soest T. (2016). Reciprocal relations between body satisfaction and self-esteem: A large 13-year prospective study of adolescents. J. Adolesc..

[9] Dooley B., Fitzgerald A., Giollabhui N.M. (2015). The risk and protective factors associated with depression and anxiety in a national sample of Irish adolescents. Ir. J. Psychol. Med..

[10] Fairburn C.G., Cooper Z. (2011). Eating disorders, DSM–5 and clinical reality. Br. J. Psychiatry.

[11] Fredrickson B.L., Roberts T.-A. (1997). Objectification Theory: Toward Understanding Women’s Lived Experiences and Mental Health Risks. Psychol. Women Q..

[12] Thompson J.K., Heinberg L.J., Altabe M., Tantleff-Dunn S. (1999). Exacting Beauty: Theory, Assessment, and Treatment of Body Image Disturbance.

[13] Knauss C., Paxton S.J., Alsaker F.D. (2008). Body Dissatisfaction in Adolescent Boys and Girls: Objectified Body Consciousness, Internalization of the Media Body Ideal and Perceived Pressure from Media. Sex Roles.

[14] Fitzsimmons-Craft E.E., Harney M.B., Koehler L.G., Danzi L.E., Riddell M.K., Bardone-Cone A.M. (2012). Explaining the relation between thin ideal internalization and body dissatisfaction among college women: The roles of social comparison and body surveillance. Body Image.

[15] Frederick D.A., Buchanan G.M., Sadehgi-Azar L., Peplau L.A., Haselton M.G., Berezovskaya A., Lipinski R.E. (2007). Desiring the muscular ideal: Men’s body satisfaction in the United States, Ukraine, and Ghana. Psychol. Men Masculinity.

[16] Robinson L., Prichard I., Nikolaidis A., Drummond C., Drummond M., Tiggemann M. (2017). Idealised media images: The effect of fitspiration imagery on body satisfaction and exercise behaviour. Body Image.

[17] Watson A., Murnen S.K., College K. (2019). Gender differences in responses to thin, athletic, and hyper-muscular idealized bodies. Body Image.

[18] Myers T.A., Ridolfi D.R., Crowther J.H., Ciesla J.A. (2012). The impact of appearance-focused social comparisons on body image disturbance in the naturalistic environment: The roles of thin-ideal internalization and feminist beliefs. Body Image.

[19] Keery H., van den Berg P., Thompson J.K. (2004). An evaluation of the Tripartite Influence Model of body dissatisfaction and eating disturbance with adolescent girls. Body Image.

[20] Karazsia B.T., Crowther J.H. (2009). Social body comparison and internalization: Mediators of social influences on men’s muscularity-oriented body dissatisfaction. Body Image.

[21] Tylka T.L. (2011). Refinement of the tripartite influence model for men: Dual body image pathways to body change behaviors. Body Image.

[22] Festinger L. (1954). A Theory of Social Comparison Processes. Hum. Relat..

[23] Schaefer L.M., Thompson J.K. (2018). The development and validation of the Physical Appearance Comparison Scale–3 (PACS-3). Psychol. Assess..

[24] Myers T.A., Crowther J.H. (2009). Social comparison as a predictor of body dissatisfaction: A meta-analytic review. J. Abnorm. Psychol..

[25] Ridolfi D.R., Myers T.A., Crowther J.H., Ciesla J.A. (2011). Do Appearance Focused Cognitive Distortions Moderate the Relationship between Social Comparisons to Peers and Media Images and Body Image Disturbance?. Sex Roles.

[26] Bailey S.D., Ricciardelli L.A. (2010). Social comparisons, appearance related comments, contingent self-esteem and their relationships with body dissatisfaction and eating disturbance among women. Eat. Behav..

[27] Fuller-Tyszkiewicz M., Chhouk J., McCann L.-A., Urbina G., Vuo H., Krug I., Ricciardelli L., Linardon J., Broadbent J., Heron K. (2019). Appearance comparison and other appearance-related influences on body dissatisfaction in everyday life. Body Image.

[28] Anson M., Veale D., Miles S. (2015). Appearance comparison in individuals with body dysmorphic disorder and controls. Body Image.

[29] Lambrou C., Veale D., Wilson G. (2012). Appearance concerns comparisons among persons with body dysmorphic disorder and nonclinical controls with and without aesthetic training. Body Image.

[30] Veale D. (2004). Advances in a cognitive behavioural model of body dysmorphic disorder. Body Image.

[31] Thompson J.K., Heinberg L.J., Tantleff S. (1991). The Physical Appearance Comparison Scale. Behav. Ther..

[32] Schaefer L.M., Thompson J.K. (2014). The development and validation of the Physical Appearance Comparison Scale-Revised (PACS-R). Eat. Behav..

[33] O’Brien K.S., Caputi P., Minto R., Peoples G., Hooper C., Kell S., Sawley E. (2009). Upward and downward physical appearance comparisons: Development of scales and examination of predictive qualities. Body Image.

[34] Liao J., Jackson T., Chen H. (2014). The structure and validity of directional measures of appearance social comparison among emerging adults in China. Body Image.

[35] Rodgers R., Chabrol H., Paxton S.J. (2011). An exploration of the tripartite influence model of body dissatisfaction and disordered eating among Australian and French college women. Body Image.

[36] Smolak L. (2004). Body image in children and adolescents: Where do we go from here?. Body Image.

[37] Oosthuizen P., Lambert T., Castle D.J. (1998). Dysmorphic concern: Prevalence and associations with clinical variables. Aust. N. Z. J. Psychiatry.

[38] Senín-Calderón C., Valdés-Díaz M., Benítez-Hernández M.M., Núñez-Gaitán M.C., Perona-Garcelán S., Martínez-Cervantes R., Rodríguez-Testal J.F. (2017). Validation of Spanish language evaluation instruments for body dysmorphic disorder and the dysmorphic concern construct. Front. Psychol..

[39] Garner D.M., Olmsted M.P., Bohr Y., Garfinkel P.E. (1982). The Eating Attitudes Test: Psychometric features and clinical correlates. Psychol. Med..

[40] Rivas T., Bersabé R., Jiménez M., Berrocal C. (2010). The Eating Attitudes Test (EAT-26): Reliability and validity in Spanish female samples. Span. J. Psychol..

[41] Baumgartner H., Homburg C. (1996). Applications of structural equation modeling in marketing and consumer research: A review. Int. J. Res. Mark..

[42] Schermelleh-Engel K., Moosbrugger H., Müller H. (2003). Evaluating the Fit of Structural Equation Models: Tests of Significance and Descriptive Goodness-of-Fit Measures. Methods Psychol. Res. Online.

[43] Cohen J. (1988). Statistical Power Analysis for the Behavioral Sciences.

[44] Cheung G.W., Rensvold R.B. (2002). Evaluating Goodness-of- Fit Indexes for Testing Measurement Invariance. Struct. Equ. Model. Multidiscip. J..

[45] Kenny D.A., Kaniskan B., McCoach D.B. (2015). The Performance of RMSEA in Models with Small Degrees of Freedom. Sociol. Methods Res..

[46] Murray S.B., Nagata J.M., Griffiths S., Calzo J.P., Brown T.A., Mitchison D., Blashill A.J., Mond J.M. (2017). The enigma of male eating disorders: A critical review and synthesis. Clin. Psychol. Rev..

[47] Badenes-Ribera L., Rubio-Aparicio M., Sánchez-Meca J., Fabris M.A., Longobardi C. (2019). The association between muscle dysmorphia and eating disorder symptomatology: A systematic review and meta-analysis. J. Behav. Addict..

[48] Boroughs M.S., Krawczyk R., Thompson J.K. (2010). Body Dysmorphic Disorder among Diverse Racial/Ethnic and Sexual Orientation Groups: Prevalence Estimates and Associated Factors. Sex Roles.

[49] Fardouly J., Pinkus R.T., Vartanian L.R. (2017). The impact of appearance comparisons made through social media, traditional media, and in person in women’s everyday lives. Body Image.

[50] Fardouly J., Vartanian L.R. (2015). Negative comparisons about one’s appearance mediate the relationship between Facebook usage and body image concerns. Body Image.

[51] Strahan E.J., Wilson A.E., Cressman K.E., Buote V.M. (2006). Comparing to perfection: How cultural norms for appearance affect social comparisons and self-image. Body Image.

[52] McLean S.A., Wertheim E.H., Marques M.D., Paxton S.J. (2019). Dismantling prevention: Comparison of outcomes following media literacy and appearance comparison modules in a randomised controlled trial. J. Health Psychol..

