# Supplementary material for: Validation of a Spanish Version of the Physical Appearance Comparison Scales
Source: Int J Environ Res Public Health. 2020 Oct 11;17(20):7399. doi: 10.3390/ijerph17207399 (PMC7600572; doi:10.3390/ijerph17207399)
Supplement: Supplementary file 1 [file ijerph-17-07399-s001.pdf]

Supplementary Material

Table S1. Descriptive statistics of items on the PACS-R (n = 1151).

Items	*Mean*	*SD*	Skewness	Kurtosis
1. When I´m out in public, I compare my physical appearance to the appearance of others.	1.98	1.09	0.07	−0.57
2. When I meet a new person (same sex), I compare my body size to his/her body size.	1.69	1.22	0.29	−0.82
3. When I´m at work or school, I compare my body shape to the body shape of others.	1.65	1.17	0.28	−0.75
4. When I´m out in public, I compare my body fat to the body fat of others.	1.38	1.22	0.58	−0.66
5. When I´m shopping for clothes, I compare my weight to the weight of others.	1.31	1.29	0.65	−0.73
6. When I´m at a party, I compare my body shape to the body shape of others.	1.60	1.27	0.33	−0.98
7. When I´m with a group of friends, I compare my weight to the weight of others.	1.26	1.11	0.71	−0.18
8. When I´m out in public, I compare my body size to the body size of others.	1.46	1.14	0.45	−0.59
9. When I´m with a group of friends, I compare my body size to the body size of others.	1.30	1.12	0.66	−0.31
10. When I´m eating in a restaurant, I compare my body fat to the body fat of others.	0.85	1.06	1.25	0.89
11. When I´m at the gym, I compare my physical appearance to the appearance of others.	2.11	1.36	−0.20	−1.13

Table S2. Descriptive statistics of items on the PACS-3 (n = 506).

Items	*Mean*	*SD*	Skewness	Kurtosis	Percentage of Affirmative Responses *
1) When I'm at a party or social gathering, I compare my overall appearance to the appearance of others.	2.97	1.13	0.04	−0.72	89.5 %
1b) When I make these comparisons, I typically believe that I look ______ than the person to whom I am comparing myself.	3.32	0.85	−0.22	−0.58	88.9%
1c) When you make these comparisons, how does it usually make you feel?	3.25	0.85	−0.03	−0.26	88.3%
2) When I'm out in public, I compare my weight/shape to the weight/shape of others.	2.75	1.14	0.23	−0.69	86.4%
2b) When I make these comparisons, I typically believe that I look ______ than the person to whom I am comparing myself.	3.34	0.86	−0.30	−0.44	84.4%
2c) When you make these comparisons, how does it usually make you feel?	3.25	0.84	−0.01	−0.34	85.4%
3) When I meet a new person (same sex), I compare my weight/shape to his/her weight/shape.	2.65	1.16	0.29	−0.73	81.6%
3b) When I make these comparisons, I typically believe that I look ______ than the person to whom I am comparing myself.	3.30	0.78	-00.11	−0.29	81.2%
3c) When you make these comparisons, how does it usually make you feel?	3.23	0.78	0.03	−0.27	81.2%
4) When I watch a movie, I compare my overall appearance to the appearance of the actors/actresses.	2.36	1.14	0.53	−0.53	73.1%
4b) When I make these comparisons, I typically believe that I look ______ than the person to whom I am comparing myself.	3.73	0.71	−0.21	−0.07	70.3%
4c) When you make these comparisons, how does it usually make you feel?	3.37	0.70	0.22	0.17	72.9%
5) When I watch television, I compare my weight/shape to the weight/shape of the actors/actresses.	2.35	1.12	0.51	−0.57	73.3%
5b) When I make these comparisons, I typically believe that I look ______ than the person to whom I am comparing myself.	3.62	0.72	−0.15	0.06	73.1%
5c) When you make these comparisons, how does it usually make you feel?	3.35	0.68	.22	00.26	73.1%
6) When I see a model in a magazine, I compare my weight/shape to his/her weight/shape.	2.42	1.21	0.53	-0.68	72.7%
6b) When I make these comparisons, I typically believe that I look ______ than the person to whom I am comparing myself.	3.71	0.75	−0.35	-0.06	68.2%
6c) When you make these comparisons, how does it usually make you feel?	3.42	0.73	0.20	0.05	72.5%
7) When I see a model in a magazine, I compare my muscularity to his/her muscularity.	2.25	1.18	0.63	−0.56	65.8%

7b) When I make these comparisons, I typically believe that I look ______ than the person to whom I am comparing myself.	3.66	0.77	−0.38	0.09	65.6%
7c) When you make these comparisons, how does it usually make you feel?	3.37	0.69	00.29	−0.02	61.1%
8) When I watch a movie, I compare my muscularity to the muscularity of the actors/actresses.	2.13	1.12	0.79	−0.19	64%
8b) When I make these comparisons, I typically believe that I look ______ than the person to whom I am comparing myself.	3.62	0.74	-0.25	0.10	63.1%
8c) When you make these comparisons, how does it usually make you feel?	3.35	0.66	0.42	0.14	60.2%
9) When I'm out in public, I compare my muscularity to the muscularity of others.	2.28	1.10	0.62	−0.28	71.1%
9b) When I make these comparisons, I typically believe that I look ______ than the person to whom I am comparing myself.	3.34	0.80	-0.22	−0.38	70.7%
9c) When you make these comparisons, how does it usually make you feel?	3.19	0.77	0.03	−0.07	70.5%

* Percentage of participants that marked response options from 2 (seldom) to 5 (almost always).
